# Spontaneous Bacterial Peritonitis Due to Bacillus licheniformis in an Autosomal Dominant Polycystic Kidney Disease Patient on Peritoneal Dialysis: A Rare Presentation Linked to Avian Exposure

**DOI:** 10.7759/cureus.68468

**Published:** 2024-09-02

**Authors:** Jawad Atrash, Ahmed Kahla, Nabil CN Khalil

**Affiliations:** 1 Internal Medicine, St. Joseph Hospital, Jerusalem, PSE; 2 Internal Medicine, Al-Makassed Hospital, Jenin, PSE

**Keywords:** bacillus licheniformis induced peritonitis, continuous ambulatory peritoneal dialysis (capd), autosomal dominant polycystic kidney disease (adpkd), peritoneal dialysis-associated peritonitis (pdap), peritoneal dialysis complication

## Abstract

This case report details a rare instance of *Bacillus licheniformis*-induced peritonitis in a 43-year-old male diagnosed with autosomal dominant polycystic kidney disease (ADPKD) undergoing peritoneal dialysis (PD). The patient presented with acute onset of severe abdominal pain and fever, prompting a microbiological investigation that revealed Gram-positive bacilli. Initial empirical treatment with ceftazidime and vancomycin was followed by targeted vancomycin therapy upon identification of *B. licheniformis*. The patient's clinical course showed steady improvement, corroborated by a recent history of avian contact. This case underscores the critical consideration of uncommon pathogens and environmental exposures in managing peritonitis among peritoneal dialysis patients.

## Introduction

*Bacillus licheniformis* is an aerobic bacterium that tends to grow facultatively anaerobically. It is a Gram-positive, rod-shaped bacterium that forms highly heat-resistant endospores and is primarily found in soil. Although most Bacillus strains, including *B. licheniformis*, are considered safe and non-pathogenic to humans [1], sporadic cases of *B. licheniformis* bacteremia have been documented in immunocompromised individuals associated with intravenous heroin products, splenectomy, malignancy, and bone marrow transplantation; fatalities are uncommon [2].

Peritonitis remains a serious complication of peritoneal dialysis (PD), leading directly to death in about 16% of PD patients and necessitating frequent hospital visits and changes in treatment methods. Prompt identification and appropriate antibiotic treatment are essential to manage peritonitis and reduce its severe effects [3]. While common causes include *Staphylococcus epidermidis* and *Staphylococcus aureus*, Gram-negative peritonitis cases, particularly from Pseudomonas species, are increasing and associated with significant health risks. Nevertheless, peritonitis caused by uncommon organisms is also documented, underscoring the diverse range of microbes responsible for this condition [4]. Reporting these less common pathogens encourages healthcare providers to consider them as potential causes of peritonitis.

Herein, we present a compelling case of peritonitis caused by *B. licheniformis*, an exceptionally rare pathogen in the context of peritoneal dialysis-associated peritonitis (PDAP). Historically documented primarily in continuous ambulatory peritoneal dialysis (CAPD) patients, our report marks the first documented occurrence of a patient with autosomal dominant polycystic kidney disease (ADPKD) who has been dependent on peritoneal dialysis for a prolonged period following an accidental encounter with birds at home.

## Case presentation

A 43-year-old male presented to the emergency department of St. Joseph Hospital in Jerusalem with severe diffuse abdominal pain and a persistent low-grade fever over the past five days. The pain was moderate in severity, non-radiating, and unaccompanied by nausea, vomiting, chills, or feverish sensations. Notably, peritoneal dialysis fluid appeared turbid without purulent discharge. There was no history of urinary symptoms, diarrhea, or prior episodes of peritonitis.

The patient had a known history of end-stage renal disease (ESRD) secondary to adult-onset polycystic kidney disease, necessitating peritoneal dialysis since 2012. He managed hypertension pharmacologically and had a history of smoking but no diabetes mellitus. On arrival, vital signs were stable, with a blood pressure of 130/74 mmHg, a pulse rate of 115 bpm, and a temperature of 38.3 °C. Physical examination revealed diffuse abdominal tenderness without rebound tenderness or guarding, and no hepatosplenomegaly was observed. Examination of other systems, including cardiovascular and respiratory, was unremarkable, with no evidence of systemic infection.

**Figure 1:**
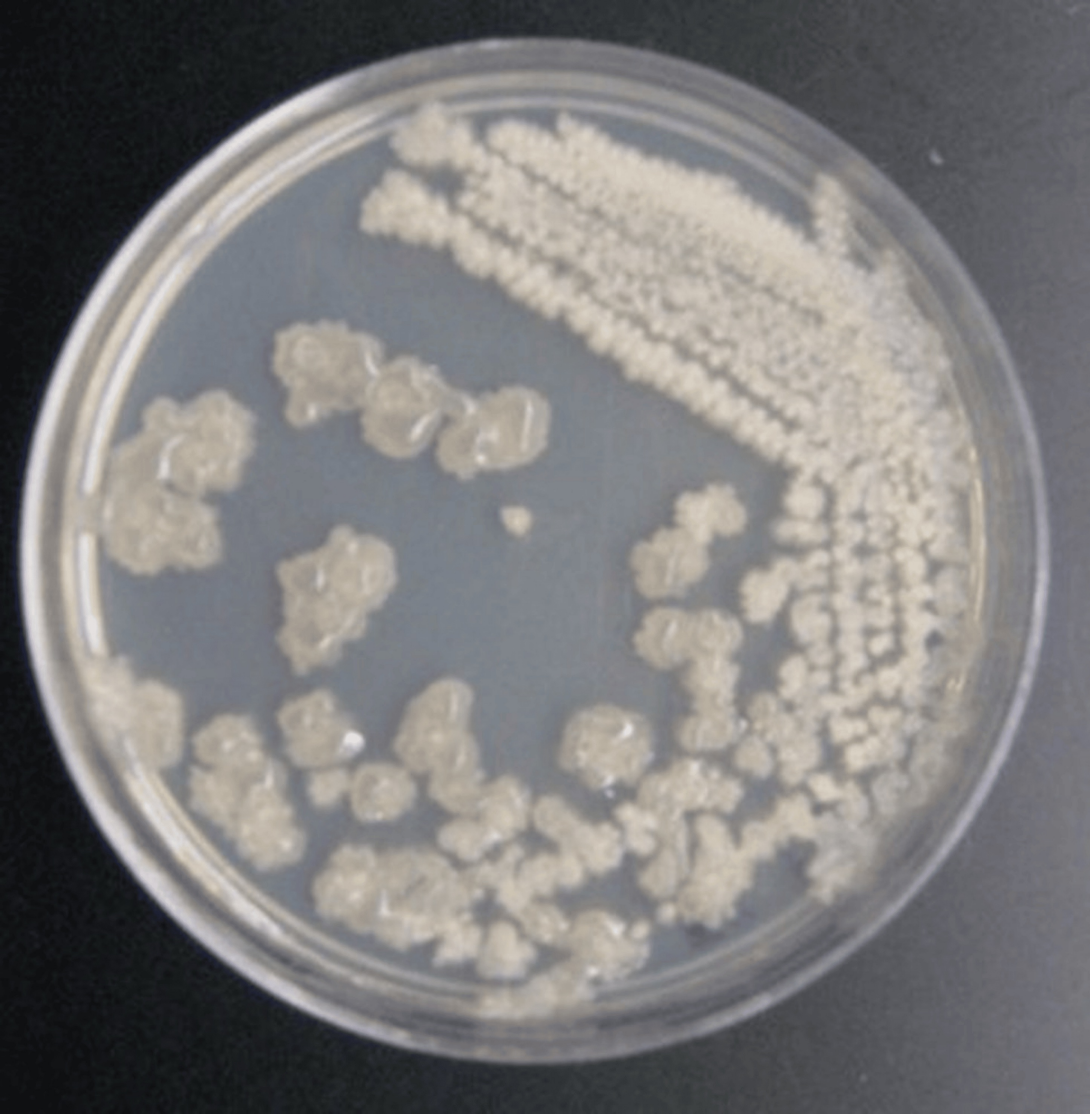
Bacillus licheniformis cultured on a blood agar plate, exhibiting Gram-positive bacilli arranged in short chains. The morphology and growth characteristics are consistent with those of B. licheniformis.

**Figure 2:**
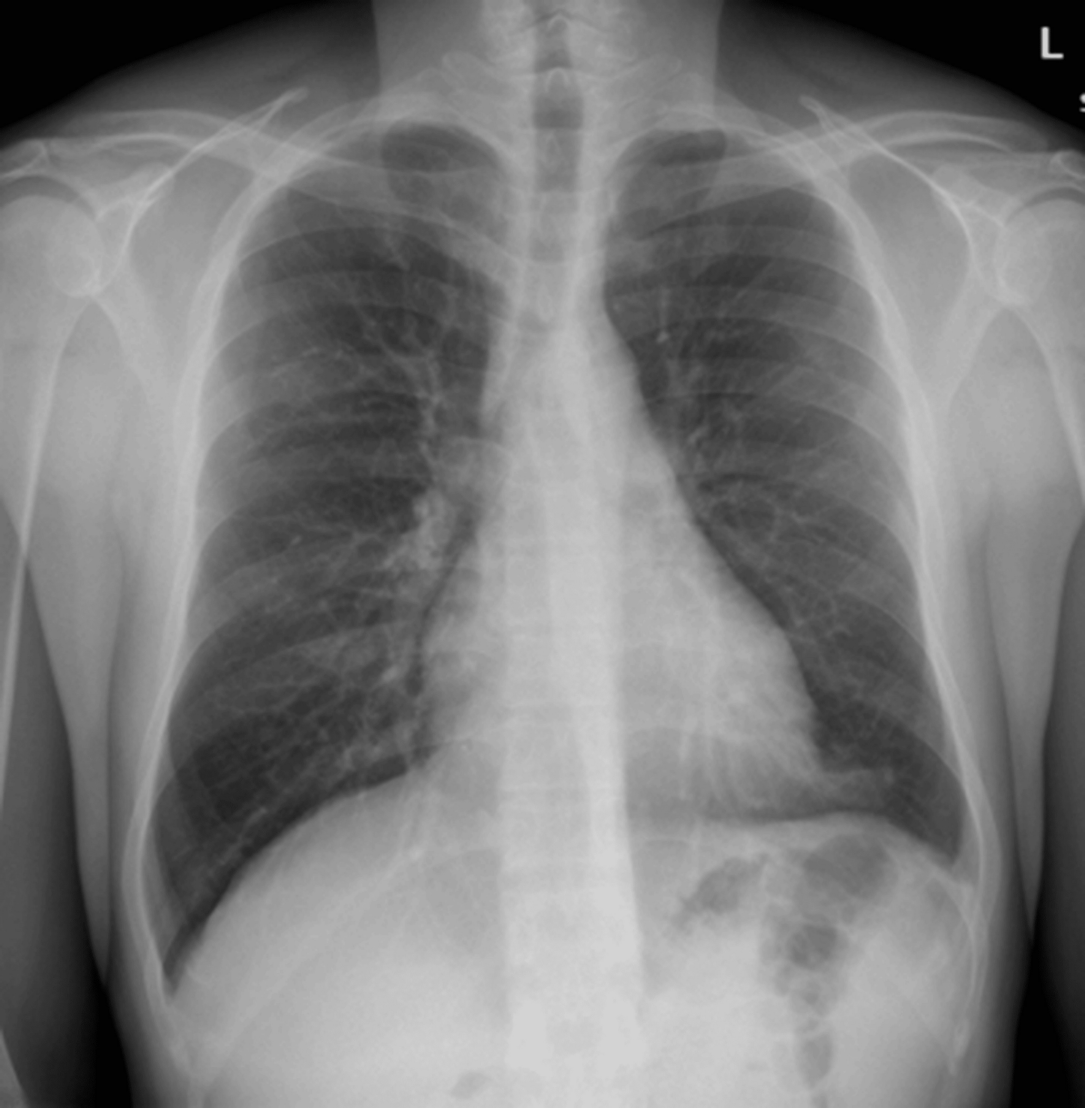
Chest X-ray (PA view), normal findings. PA: posterior anterior.

Initial laboratory investigations showed no leukocytosis, anemia, or thrombocytopenia. Renal and liver function tests returned normal results. Peritoneal fluid was aspirated at admission, and microbiological analysis was performed alongside blood cultures. Gram stain of the sample revealed abundant pus cells and Gram-positive bacilli in short chains (Figure 1). Chest X-ray results ruled out pulmonary complications (Figure 2).

Given clinical suspicion of peritonitis and confirmed peritoneal dialysis fluid analysis showing a white blood cell count >200, empirical antibiotic therapy was promptly initiated with intravenous ceftazidime 1 g every eight hours and vancomycin 2 g every 12 hours pending culture results. The patient responded positively to therapy, with reduced abdominal pain and stabilized vital signs within the first 24 hours.

After five days of therapy initiation, repeat aspiration of peritoneal fluid yielded no bacterial growth on the same media. However, subsequent culture results from both blood and peritoneal fluid identified Bacillus species, specifically *B. licheniformis*. Ceftazidime was discontinued upon identification of the organism's susceptibility profile, confirming susceptibility to vancomycin. The patient's antibiotic regimen was adjusted accordingly to vancomycin 1 g intravenously every 12 hours.

Upon further inquiry, the patient disclosed recent exposure to birds at home. Clinical monitoring over the following days demonstrated sustained improvement in symptoms with resolution of abdominal tenderness. The patient's hospital course remained uncomplicated, without recurrence of symptoms or signs of systemic infection. He completed a 14-day course of vancomycin without adverse effects and was discharged in stable condition with scheduled outpatient follow-up. Our recommendation for the patient is to avoid exposure to birds as a preventive measure. Fortunately, as of the latest follow-up, the patient has not experienced any recurrence of peritonitis.

## Discussion

Peritonitis remains a leading cause of transition from PD to hemodialysis, contributing significantly to morbidity and mortality rates ranging from 3.5% to 10% [5]. Despite adherence to appropriate antibiotic regimens, peritonitis often recurs, necessitating catheter removal. The recurrence is frequently associated with biofilm formation on PD catheters [6], a phenomenon notably absent in our case, where the patient achieved successful treatment without relapse.

The absence of biofilm-associated recurrence underscores the effectiveness of the chosen antibiotic therapy and the critical role of timely intervention. Successful resolution of peritonitis without catheter removal highlights the potential benefits of early and targeted antibiotic therapy in preventing complications and maintaining PD efficacy.

PD-related peritonitis poses significant risks of morbidity and mortality, with multifaceted etiologies including contamination from the skin, the transmission of intestinal bacteria via the bowels, or from blood sources [7]. Preventive measures against peritonitis include meticulous exit-site care, effective management of exit-site infections, thorough hand hygiene practices, comprehensive patient education, avoidance of constipation, and adherence to the flush-before-fill technique [8].

Zoonotic peritonitis, particularly from close contact with companion animals, is well documented in the literature. Data from the French-speaking registry for peritoneal dialysis (RDPLF) indicate that zoonotic infections represent 0.54% of all cases of PD-related peritonitis in Western European countries, with a notable mortality rate of 13.5% [9]. In 27% of reported cases, catheter removal was necessary, underscoring the severity and complexity of these infections [10].

However, there is a dearth of reported cases among Middle Eastern and Arab populations. Further research and reporting are imperative to better understand the prevalence, clinical presentation, and outcomes of zoonotic peritonitis in these demographic groups.

The diversity of potential zoonotic pathogens complicates the development of standardized antibiotic guidelines. Therefore, accurate diagnosis through a positive culture of peritoneal effluent remains pivotal for initiating appropriate antibiotic therapy [11]. Clinicians must maintain a high index of suspicion for zoonotic microorganisms when patients have a history of close animal contact or engage in animal-related occupations, as these factors may indicate the underlying cause of peritonitis.

Structured training sessions, educational initiatives, and hygiene counseling directed at patients and their companions who interact with domestic animals have proven effective in mitigating the incidence and recurrence of pet-associated peritonitis, as demonstrated by Broughton et al. [12]. These interventions include implementing barriers to prevent pets from accessing dialysis equipment, promoting rigorous hand hygiene practices, and educating caregivers and patients about the risks of zoonotic infections. Such proactive measures not only enhance patient safety but also underscore the importance of a multidisciplinary approach in managing complications related to peritoneal dialysis [12].

Integration of these strategies into clinical practice can substantially reduce the burden of pet-associated peritonitis, thereby improving treatment outcomes and overall quality of care for peritoneal dialysis patients.

## Conclusions

To date, literature reports have documented three cases of *B. licheniformis* infection, all occurring post-diarrhea. One case exhibited relapse, while another was successfully eradicated following a complete two-week course of intraperitoneal vancomycin treatment. In our case, although the causative organism is rare, a thorough investigation into contact, behavioral, and environmental history remains imperative and helpful.

## References

[1] Baumann P, Clark MA, Baumann L, Broadwell AH (1991). Bacillus sphaericus as a mosquito pathogen: properties of the organism and its toxins. Microbiol Rev.

[2] Haydushka IA, Markova N, Kirina V, Atanassova M (2012). Recurrent sepsis due to bacillus licheniformis. J Glob Infect Dis.

[3] Segal JH, Messana JM (2013). Prevention of peritonitis in peritoneal dialysis. Semin Dial.

[4] Li PK, Szeto CC, Piraino B (2016). ISPD peritonitis recommendations: 2016 update on prevention and treatment. Perit Dial Int.

[5] Al Sahlawi M, Perl J (2022). Peritoneal dialysis peritonitis outcomes: getting to the heart of the matter. Kidney Int Rep.

[6] Burke M, Hawley CM, Badve SV (2011). Relapsing and recurrent peritoneal dialysis-associated peritonitis: a multicenter registry study. Am J Kidney Dis.

[7] Salzer WL (2018). Peritoneal dialysis-related peritonitis: challenges and solutions. Int J Nephrol Renovasc Dis.

[8] Gursu M, Shehaj L, Elcioglu OC, Kazancioglu R (2023). The optimization of peritoneal dialysis training in long-term. Front Nephrol.

[9] Abebe M, Laveglia C, George S, Wadhwa NK (2014). Pet-related peritonitis and its prevention in peritoneal dialysis: a case study. Perit Dial Int.

[10] Mihalache O, Doran H, Catrina E, Bobircă F, Mustatea P, Georgescu D, Pătrașcu T (20147). Diagnosis characteristics and therapeutical options of infectious complications associated with peritoneal dialysis. J Med Life.

[11] Li PK, Chow KM, Cho Y (2022). ISPD peritonitis guideline recommendations: 2022 update on prevention and treatment. Perit Dial Int.

[12] Broughton A, Verger C, Goffin E (2010). Pets-related peritonitis in peritoneal dialysis: companion animals or trojan horses?. Semin Dial.

